# Induction of colon and cervical cancer cell death by cinnamic acid derivatives is mediated through the inhibition of Histone Deacetylases (HDAC)

**DOI:** 10.1371/journal.pone.0186208

**Published:** 2017-11-30

**Authors:** Preethi G. Anantharaju, Deepa B. Reddy, Mahesh A. Padukudru, CH. M. Kumari Chitturi, Manjunath G. Vimalambike, SubbaRao V. Madhunapantula

**Affiliations:** 1 Department of Biochemistry, Center of Excellence in Molecular Biology and Regenerative Medicine, JSS Medical College, Jagadguru Sri Shivarathreeshwara University,Mysuru, Karnataka, India; 2 Department of Applied Microbiology, Sri Padmavati Mahila Visvavidyalayam (Women’s University), Tirupati, Andhra Pradesh, India; 3 Department of Pulmonary Medicine, JSS Medical College, Jagadguru Sri Shivarathreeshwara University, Mysuru, Karnataka, India; 4 Department of Pathology, JSS Medical College, Jagadguru Sri Shivarathreeshwara University, Mysuru, Karnataka, India; University of Navarra, SPAIN

## Abstract

Recent studies from our group and many others have shown the ability of histone deacetylase (HDAC) inhibitors for retarding the growth of carcinomas of cervix, colon and rectum in vitro. A search for naturally occurring HDAC inhibitors continues due to the adverse effects associated with known HDAC inhibitors like SAHA and TSA. Therefore in the current study, naturally occurring cinnamic acids derivatives were screened for HDAC inhibitory effect using in silico docking method which identified cinnamic acids as potential candidates. Cinnamic acids (CA) are naturally occurring phenolic compounds known to exhibit anticancer properties. However, it is not clearly known whether the anticancer properties of CA derivatives are due to the inhibition of oncogenic HDACs, if so how the efficacy varies among various CA derivatives. Hence, the HDAC inhibitory potential of CA derivatives containing increasing number of hydroxylic groups or methoxy moieties was determined using Discovery Studio software and the most potent CA derivatives tested ex vivo (biochemical assay) as well as in vitro (using cell based assay). Among CA derivatives tested, dihydroxy cinnamic acid (DHCA, commonly known as caffeic acid) exhibited better interactions with HDAC2 (compared to other isoforms) in silico and inhibited its activity ex vivo as well as in vitro. Targeted reduction of HDAC activity using DHCA induced death of cancer cells by (a) generating reactive oxygen species, (b) arresting cells in S and G2/M phases; and (c) induction of caspase-3 mediated apoptosis. In conclusion, we demonstrated that DHCA inhibited cancer cell growth by binding to HDAC followed by the induction of apoptosis.

## Introduction

Histones are DNA bound proteins, which forms the chromatin. When post translationally modified (by acetylation), they regulate the expression of underlying genes [1]. Histones undergo acetylation and deacetylation, respectively, by Histone Acetyl Transferases (HATs) and Histone De-Acetylases (HDACs) [2]. Whereas HATs increase apoptosis in cells by transcriptional activation of genes such as p53 and Rb, the HDACs promotes cancer progression not only by silencing the expression of tumor suppressor proteins but also by triggering reactive oxygen species (ROS) [3]. Among various HDACs, the HDAC1, HDAC2, HDAC3 and HDAC4 are known to promote cancer cell growth by inhibiting the expression of cell cycle regulators p21 and proapoptotic Bax proteins [4,5]. In addition to deacetylate histone proteins, HDAC’s can also act on non-histone proteins that are involved in the regulation of cell cycle, differentiation and apoptosis [6]. For instance, interaction of HDAC1 with P53, deactivates its tumor suppressor activity [7]. Therefore, inhibiting HDACs’ activity is a viable strategy to retard cancer cells growth [8]. Supporting this statement, inhibitors of HDAC such as SAHA and TSA have shown promising results in vitro as well as in preclinical animal models[9,10]. Few HDAC inhibitors have also been tested in clinical trials for inhibiting cancers [11]. For example, vorinostat and romidepsin have been evaluated for the treatment of cutaneous T-cell lymphoma (CTCL) [11]. Likewise, belinostat was approved for the treatment of peripheral T-cell lymphoma (PTCL) [12]. However, the success of these compounds as monotherapies was minimal [13], hence subsequent studies have evaluated these agents in combination [14]. A recent study by Fenichel, M.P. et al., 2015 has tested panobinostat in combination with the proteasome inhibitor bortezomib. This combination was approved for refractory multiple myeloma [14]. While responses to single- agent HDACi are limited in solid tumors [15], studies in non-small cell lung cancer and estrogen receptor-positive advanced breast cancer suggest they may have efficacy in combination therapy regimens ([16];[17]). However, to date, no confirmatory data in large clinical trials was available about the efficacy of HDAC inhibitors (alone or in combination) for treating advanced metastatic tumors [18,19]. Hence, the need to identify a potent naturally occurring HDAC inhibitor still prevails. A recent study from our laboratory screened and identified benzoic acid derivatives as the inhibitors of HDACs [20]. But, it is not known whether the predominant cell wall phenolic compounds viz., the derivatives of cinnamic acids, also inhibit HDACs, if so, what are the structural determinants of cinnamic acid derivatives responsible for HDAC inhibition. Therefore, a screen was conducted to identify potent cinnamic acid derivatives that inhibit HDACs using in silico approach [21].

Cinnamic acids are phenolic compounds present in plant cell walls either in free- or bound form [22]. Chemically these compounds contain a propionic acid side chain attached to an aromatic ring[23] [24]. Cinnamic acid derivatives such as p-coumaric acid, ferulic acid, sinapic acid, caffeic acid are well explored plant phenolic compounds commonly found in fruits and vegetables [25]. A study by Waldecker et al in 2008 demonstrated, for the first time, the HDAC inhibitory activity of p-coumaric acid and caffeic acid in colon cancer cells [26]. Subsequently, a separate study by Bora-Tartar et al in 2009 had shown that other cinnamic acid derivatives such as chlorogenic acid also inhibit HDAC activity [27]. However, the key structural features required for exhibiting potent HDAC inhibitory activity of cinnamic acids was not studied. Therefore, in the present investigation, cinnamic acid derivatives with increasing–OH groups and–OCH_3_ groups have been tested for exhibiting HDAC inhibitory effects using in silico docking studies. Next, the potent compound exhibiting stronger binding to HDAC was evaluated for inhibiting HDAC protein mixture present in the nuclear extract of HeLa. Results of these experiments identified DHCA (Caffeic acid) as the potent HDAC inhibitor, hence, it was tested for retarding cancer cell growth (in vitro) as well as for down modulating the HDAC activity. In addition, the mechanisms of action of DHCA for inhibiting cancer cell growth were determined by measuring the levels of apoptosis using acridine orange and ethidium bromide staining, as well as by assessing the levels of caspase-3 expression.

## Materials and methods

### Materials

Cinnamic acid, derivatives of cinnamic acid (HCA, DHCA, MHMMCA, DMCA, MHDMCA and TMCA) were procured from Sigma Aldrich, St. Louis, MO, USA.

Table 1 shows the abbreviated name, common- and IUPAC names and molar mass of CA derivatives used for screening HDAC inhibitory activity.

**Table 1:** Common- and IUPAC name of CA derivatives.

Abbreviated Name	Common Name	IUPAC Name	Molar Mass (g/mol)
Cinnamic acid (CA)	Cinnamylic acid	3-phenylprop-2-enoic acid	148.16
Hydroxy Cinnamic acid (HCA)	*p-*Coumaric acid	3-(4-hydroxyphenyl)-2-propenoic acid	164.16
Dihydroxy Cinnamic acid (DHCA)	Caffeic acid	3-(3,4-Dihydroxyphenyl)-2-propenoic acid	180.16
Monohydroxy, Monomethoxy Cinnamic acid (MHMMCA)	Ferulic acid	3-(4-hydroxy-3-methoxy-phenyl)prop-2-enoic acid	194.18
Dimethoxy Cinnamic acid (DMCA)	Caffeic acid diethyl ether	3-(3,4-Dimethoxyphenyl)-2-propenoic Acid	208.21
Monohydroxy, Dimethoxy Cinnamic acid (MHDMCA)	Sinapinic acid	3-(4-hydroxy-3,5-dimethoxyphenyl)prop-2-enoic acid	224.21
Trimethoxy Cinnamic acid (TMCA)	Trimethoxy Cinnamic acid	3-(3,4,5-trimethoxyphenyl) 2-Propenoic acid	238.24

Cancer cell lines representing carcinomas of cervix (HeLa and SiHa), and colon and rectum (HCT-116 and HCT-15) were procured from National Centre for Cell Sciences (NCCS), Pune, Maharashtra, India. NCCS is an autonomous institute of Department of Biotechnology (DBT), Government of India serving as a national repository for providing characterized cell lines. For additional information about NCCS please visit http://www.nccs.res.in/. Cell culture reagents and disposables for cell culture were procured from Life Technologies, Carlsbad, CA, USA and Tarsons India Pvt Ltd, Mumbai, Maharashtra, India, respectively. Histone deacetylase fluorometric (HDAC) assay kit [cat#: ab156064] was from Abcam, Cambridge, UK. Bicinchoninic acid (BCA) protein estimation kit [cat#: 23227] was from Thermo Fischer Scientific, Waltham, MA, USA. 2',7'-dichlorodihydrofluorescein diacetate (H2DCF-DA) was from Sigma Aldrich, St. Louis, USA. Lactate dehydrogenase activity assay kit [cat#: K730-500] and Caspase-3/CPP32 colorimetric assay kit [cat#: K106-100] were from Biovision Milpitas, CA, USA. Acridine orange, ethidium bromide, propidium iodide were from SRL, Mumbai, Maharashtra, India. Accelrys Discovery Studio 3.5 software was from Biovia, San Diego, CA, USA (Licensed to Sri Padmavati Mahila Visvavidyalayam (Women’s University).

## Methods

### Comparative assessment of the binding potential of cinnamic acid and cinnamic acid derivatives to Trichostatin-A binding site of HDAC using Discovery Studio 3.5

#### Selection of HDAC structure from protein data bank

Comparative assessment of the binding potential of cinnamic acid and cinnamic acid derivatives was evaluated for Class-1 HDAC enzymes (HDAC 1, HDAC 2, HDAC3 and HDAC8) to the Trichostatin-A binding site. The X-ray structures of human HDAC1 in complex with dimeric ELM2-SANT domain of MTA 1 from the NuRD complex (PDB ID:4BKX) [28], HDAC2 (one of the predominant form, over expressed in cancers, among different HDACs) complexed with N-(2-aminophenyl) benzamide (PDB ID:3 MAX) [29], HDAC3 bound to co-repressor an inositol tetraphosphate (PDB ID:4A69) [30], HDAC8 with inhibitor complex (PDB ID: 2V5X) [31] and HDAC-4 with hydroxamic acid inhibitor (PDB ID:2VQV) was retrieved from Research Collaboratory for Structural Bioinformatics (RCSB) Protein Data Bank (http://www.rcsb.org/pdb) as described in our recent publication [20]. The PDB files were cleaned and the hetero-atoms (HETATM) of the receptors removed manually as they were the non-standard residues of proteins [29]. Prior to docking, the proteins were prepared using “Prepare Protein” module available in Discovery Studio.

#### Generation of ligand dataset

Ligand structures and the properties (hydrogen donors, acceptors, Log-P values, refractivity, pH and molecular weight) of each ligand were extracted from PubChem compound database (https://pubchem.ncbi.nlm.nih.gov/) [32]. Before docking, the ligands were prepared using the ‘‘prepare ligand” module (available in Discovery studio 3.5) for (a) removing duplicates; (b) enumerating isomers and tautomers; and (c) generating 3D conformations [32].

#### Active site analysis of HDAC Isoforms

Possible binding sites of HDAC were identified and screened based on the literature data, PDB site records and receptor cavities analysis using binding site tools available in Discovery Studio (18, 19, 20, 21). Among the various binding sites evaluated, the site which demonstrated lowest binding energy with Trichostatin A (control) was considered for further evaluation with other compounds. The CA derivative which exhibited potent interactions with HDAC at TSA binding sites were also docked at SAHA and sodium butyrate binding site.

#### Molecular docking using Discovery Studio 3.5

C-docker module was used for the docking of cinnamic acid and cinnamic acid derivatives in the receptor-binding sit [33]. Specific CDOCKER scores for different poses of protein-ligand complex were obtained after docking. The best ligand with lower C docker energy scores and higher number of interacting amino acid residues were selected.

### Ex vivo and in vitro assessment of HDAC inhibitory potential of cinnamic acid and cinnamic acid derivatives

Histone deacetylase (HDAC) inhibitory potential of CA and CA-derivatives was determined using the nuclear extract (of HeLa cells) supplied in the fluorometric HDAC assay kit (ab156064; Abcam, Cambridge, UK). In addition, the nuclear extract of HCT-116 cell line treated with CA derivatives (In vitro analysis) was also analyzed for HDAC inhibitory effects. The kit is designed to measure the activity of Class I HDACs in cell- and tissue protein extracts. In principle, the kit works by coupling HDAC reaction with an easy-to-measure peptidase reaction, which estimates the amount of released 4-amino-7-methylcoumarin (AMC).

#### Isolation of cellular nuclei and collection of nuclear extract

The nuclear fraction of cells were isolated as per the kits protocol (ab156064; Abcam, Cambridge, UK). In brief, 1 x 10^6^ untreated, vehicle treated and compound (CA derivative) exposed cells were trypsinized and pelleted at 3000rpm for 5 minutes and washed thrice with PBS. The pelleted cells were resuspended in 1.0mL lysis buffer containing 10mM Tris HCl (pH 7.5), 10mM NaCl, 15mM MgCl_2_, 250mM Sucrose, 0.5% NP-40 and 0.1mM EGTA. The samples were mixed thoroughly and incubated on ice for 15.0 minutes, and later centrifuged at 10,000rpm for 10.0 minutes at 4°C with 4.0mL of sucrose cushion. The collected pellet was washed using cold 10.0mM Tris HCl (pH 7.5) and 10.0mM NaCl to obtain a nuclei rich fraction.

#### Extraction of nuclei

The nuclei rich fraction was resuspended in ~100.0μL nuclear extraction buffer containing 50mM HEPES-KOH (pH 7.5), 420mM NaCl, 0.5mM Na_2_EDTA, 0.1mM EGTA, 10% Glycerol. The mixture was vortexed and incubated for 30.0 minutes on ice and centrifuged at 20,000rpm for 10 minutes. The supernatant (crude nuclear extract) was used for determination of HDAC activity. The protein estimation of nuclear extract was carried out using BCA kit (Thermo Fischer Scientific, Waltham, MA, USA) as described below and extracts stored at -80°C until further use.

#### Estimation of total protein using BCA method

Pierce BCA kit from Thermo Fischer Scientific was used for estimation of total protein content in the nuclear extracts [34] [https://tools.thermofisher.com/content/sfs/manuals/MAN0011430_Pierce_BCA_Protein_Asy_UG.pdf]. 10.0μL of increasing concentration of 25, 125, 250, 500, 750, 1000 and 1500μg/mL of bovine serum albumin (BSA) was incubated with 200μL BCA reaction mixture (containing 50 parts of reagent A containing 0.8% sodium bicarbonate, 4% bicinchoninic acid and 0.16% sodium tartrate in 0.1M sodium hydroxide and 1 part of reagent B made up of 4% cupric sulfate) for 30.0minutes at 37°C, and read at 562nm using a multimode plate reader (PerkinElmer, Waltham, MA, USA). Appropriately diluted test samples were also incubated similarly and concentration calculated using the standard calibration curve.

#### HDAC inhibitory potential assessment using ex vivo method

HDAC inhibitory potential of CA and CA-derivatives was determined using HeLa crude nuclear extract provided in the abcam HDAC kit. Experimentally, 5.0μL assay buffer and 5.0μL fluoro-substrate were mixed with 5.0μL of HeLa nuclear extracts and the reaction volume adjusted to 30.0μL using distilled water and incubated for 20.0minutes at room temperature. Later 20.0μL of stop solution was added to stop the reaction followed by addition of 5.0μL of developer. The fluorescence was read using kinetic mode at an excitation of 365nm and emission of 450nm. Appropriate experimental controls that include (a) no enzyme control with all components except the nuclear lysates; (b) a developer control consisting of fluro-deacetylated substrate instead of fluoro-substrate; (c) an inhibitor control with 10.0μM of Tricostatin-A (a known HDAC Inhibitor); and (d) a solvent control were also processed similarly.

#### HDAC inhibitory potential assessment using in vitro method

In vitro HDAC inhibitory potential assessment was carried out as described by Senawoong T et al., 2013 [35] using colon cancer cells. Experimentally, first, 1 x 10^6^ exponentially growing HCT-116 cells were treated with DHCA (250μM, 750μM and 1500μM), and Tricostatin A (10.0μM) for 48h. The cells were trypsinized, and nuclear lysates collected (10.0μg) were used for estimation of HDAC enzyme activity as detailed before.

### Determination of the anti-cancer activity of cinnamic acid derivatives

The anti-cancer activity of cinnamic acid derivatives was measured according to Madhunapantula et al., 2008 [36]. 0.5 x 10^4^ colon (HCT-116 and HCT-15) and cervical (HeLa and SiHa) cancer cells, in 100μL DMEM supplemented with 10% FBS were seeded in 96-well plates and incubated for 48h at 37°C in a cell culture incubator maintained at 5% CO_2_ and 95% relative humidity. The cells were treated with increasing concentration of cinnamic acid derivatives (dissolved in DMSO and diluted in DMEM-10% FBS medium) for 24h, 48h and 72h. Percentage viability compared to vehicle DMSO treated cells, was measured using sulforhodamine-B assay.

#### Measurement of cell viability using sulforhodamine-B assay (SRB assay)

SRB assay was performed according to Skehan et al., 1990 [37]. Experimentally, cells were fixed with 50.0μL of cold 50% (w/v) TCA for 1hour at 4°C. Later, the wells were washed with water (200μL X 4 times) to remove TCA and serum proteins. The plates were air dried and incubated with 100μL 0.4% SRB (prepared in 1% acetic acid) for 30.0minutes to stain the cellular proteins. The plates were washed with 1% acetic acid (200μL) for 3 to 4 times to remove the unbound SRB. 10.0mM Tris base solution (100μL/well) was used to solubilize the bound SRB and the absorbance measured in a multimode plate reader operating at 490nm. Percentage cell viability calculated using the equation shown below:

$$\%\;Viability=100-\left[\{\frac{\left(OD\;of\;control-OD\;of\;sample\right)}{\left(OD\;of\;control\right)}\}x\;100\right]$$

#### Determination of cell viability by assessing the levels of lactate dehydrogenase release in to the cell culture media

Amount of LDH released in to the medium from the colon cancer cells treated with DHCA was measured using Picoprobe lactate dehydrogenase activity assay kit from Biovision. (http://www.biovision.com/manuals/K730.pdf). Lactate dehydrogenase, a marker of tissue damage catalyzes the conversion of lactate to pyruvate. The extent of damage caused by the compounds to the cells can be directly correlated to the amount of LDH released in to the media. First, 0.3 x 10^6^ HCT-116 cells in 2.0mL media/well were plated in a 6-well plate. Next, after 48h, the exponentially growing cells were treated with 250μM, 750μM and 1500μM of DHCA for 48h. Twenty five micro liters media was collected from the treated cells and made up to 50.0μL with LDH assay buffer. Next, 50.0μL reaction mixture containing 45.5μL of LDH assay buffer, 2.5μL of Picoprobe and 2.0μL LDH substrate was added. The fluorescence was measured immediately at 37°C using a multimode plate reader at an excitation of 535nm and emission of 587nm.

### Evaluation of intracellular reactive oxygen species (ROS)

Levels of ROS was estimated according to Shailasree et al., 2015 with minor modifications [38]. 0.5 x 10^4^ HCT-116 cells / well were plated in 96 well plates and incubated in a carbon dioxide incubator (maintained at 5% CO_2_) at 37°C for 48h. The growing cells were first treated with DHCA (0.312, 0.625, 1.25, 2.5, 5.0mM) for 24h and 48h, and subsequently with 250.0μM H_2_O_2_ for 1h. Cells were washed with PBS to remove the traces of media and incubated with 10.0μM 2', 7’-dichlorodihydrofluorescein diacetate (H2DCFDA, prepared in PBS). The plates were wrapped with sterile aluminum foil and incubated at 37°C in the CO_2_ incubator for 30 minutes. Amount of fluorescence was measured using a multimode plate reader operating at an excitation of 435nm and emission of 520nm.

### Cell cycle analysis using propidium iodide staining

Cell cycle analysis was carried out according to Hrgovic et al., 2016 [39] [http://www.abcam.com/protocols/flow-cytometric-analysis-of-cell-cycle-with-propidium-iodide-dna-staining]. 0.3 X 10^6^ HCT-116 cells were plated in a 6-well plate and allowed to reach ~70% confluence. Growing cells were treated with DHCA at 250μM, 750μM, and 1500μM concentrations for 48h. Cells treated with 50.0μM oxaliplatin (for 48h) were used as positive control. Control and treated cells were collected by trypsinization, washed thrice with PBS and fixed with chilled 70% ethanol for half an hour at -20°C. The samples were then centrifuged to remove ethanol and washed twice with PBS. The cells pelleted were incubated with 400μL propidium iodide (50μg/mL) for 15 minutes and analyzed using BD-FACS caliber. A total of 8000 cells were counted and histograms gated based on the distribution of cells in control untreated samples.

### Measurement of p21 expression in colorectal cancer cell lines treated with CA derivatives using western blotting

In order to determine whether inhibition of HDAC activity using DHCA also trigger the expression of p21, 2 x 10^6^ HCT-116 cells were (in p-100 plates) treated with DHCA (250, 750 and 1500μM) for 48h and total cells lysed using RIPA buffer consisting of 20mM Tris HCl (pH 7.5), 150mM NaCl, 1mM Na_2_EDTA, 1mM EGTA, 1% NP-40, 1% Sodium deoxycholate, 2.5mM Sodium pyrophosphate, 1mM β-glycerophosphate, 1mM activated sodium orthovanadate and 1.0μg/mL leupeptin. The cell lysates collected from untreated-, vehicle 1%DMSO treated- and DHCA exposed cells were centrifuged at 14000rpm for 15 minutes at 4°C and the supernatant separated. Total protein content in the supernatants was determined using BCA. 100μg total protein/sample was mixed with 4X sample buffer and 50mM DTT, and denaturation and complexation (with SDS) of proteins carried out by heating at 85°C for 10 minutes. The proteins were separated by NuPAGE 10% BIS-TRIS PAGE gels and subsequently transferred to PVDF. The membranes were blocked using 3% Bovine Serum Albumin in TBST at room temperature and probed with ERK-2 and p21 primary antibodies (1:2000 dilutions) from Santa Cruz Biotechnology (Santa Cruz, CA). After 12h of incubation at 4°C, the membranes were washed with TBST (Tris Buffered Saline with Tween (0.1%)) thrice and probed with secondary antibody conjugated with horseradish peroxidase from Santa Cruz Biotechnology (Santa Cruz, CA). The blots were washed with TBST and developed using enhanced chemiluminescence reagent from Invitrogen, Carlsbad, CA.

### Detection of apoptosis by acridine orange and ethidium bromide staining

Apoptosis detection using acridine orange and ethidium bromide staining method was carried out as described by Shailasree et al., 2015 [38]. In brief, 0.3 x 10^6^ cells were plated in 6-well plates and after ~36h exposed to increasing concentrations of DHCA (250μM, 750μM, 1500μM) and oxaliplatin (50μM) for about 48h. The control and treated cells were trypsinized and mixed thoroughly to obtain a single cell suspension. Trypsin was neutralized by the addition of complete medium (4.0mL) and 20.0μL cell suspension was incubated with 10.0μL ethidium bromide (100.0μg/mL in PBS) and 10.0μL acridine orange (100.0μg/mL in PBS) mixture for 5.0 minutes. The cells were imaged using fluorescence microscope with TRITC and FITC filters. The images obtained using 2 different channels were later merged to obtain a combined image, which showed green (live) and orange (apoptotic) cells. At least 5.0 different fields were considered for quantifying the live and apoptotic cells and the percentage cells undergoing apoptosis measured [38].

### Confirmation of apoptosis by measuring caspase-3 activity using a chromogen-coupled DEVD substrate

Activation of caspase-3 is an indicator of apoptosis in mammalian cells [40]. The caspase-3/CPP32 colorimetric assay kit, which was used to measure the caspase-3 activity [http://www.biovision.com/manuals/K106.pdf] estimates the levels of caspase-3 in cells. Experimentally, first, 0.5 x 10^6^ HCT-116 cells were treated with DHCA (250μM, 750μM, 1500μM) for 48h. While cells treated with 50.0μM Oxaliplatin served as positive control the cells exposed to 1% DMSO provided the values for vehicle control. Cell lysates from control and treated cells were collected using 100μL of lysis buffer provided in the kit. Total protein content in the cell lysates was estimated using BCA method as described earlier. Caspase-3 activity was determined by incubating 100.0μg of total protein in a total volume of 50.0μL cell lysis buffer with 50,0μL of 2X reaction buffer containing 10.0mM DTT and 5.0μL 4.0mM DEVD-pNA substrate (200.0μM final concentration) at 37°C for 3h. The developed color was read at 405nm using a multimode plate reader. The fold change compared to vehicle DMSO treated cells was calculated and plotted against compound concentration.

### Statistical analysis

All experiments were conducted with at least 3-replicates and the results of 3 such independent experiments expressed as mean + SEM. The data was subjected to one-way analysis of variance (One-Way ANOVA), followed by Tukey’s post hoc test to determine the difference between DHCA and controls/positive controls. A “P” value of < 0.05 was considered to be significant.

## Results

### Cinnamic acid derivatives effectively bind to TSA-binding site of human HDAC

**Fig 1:**
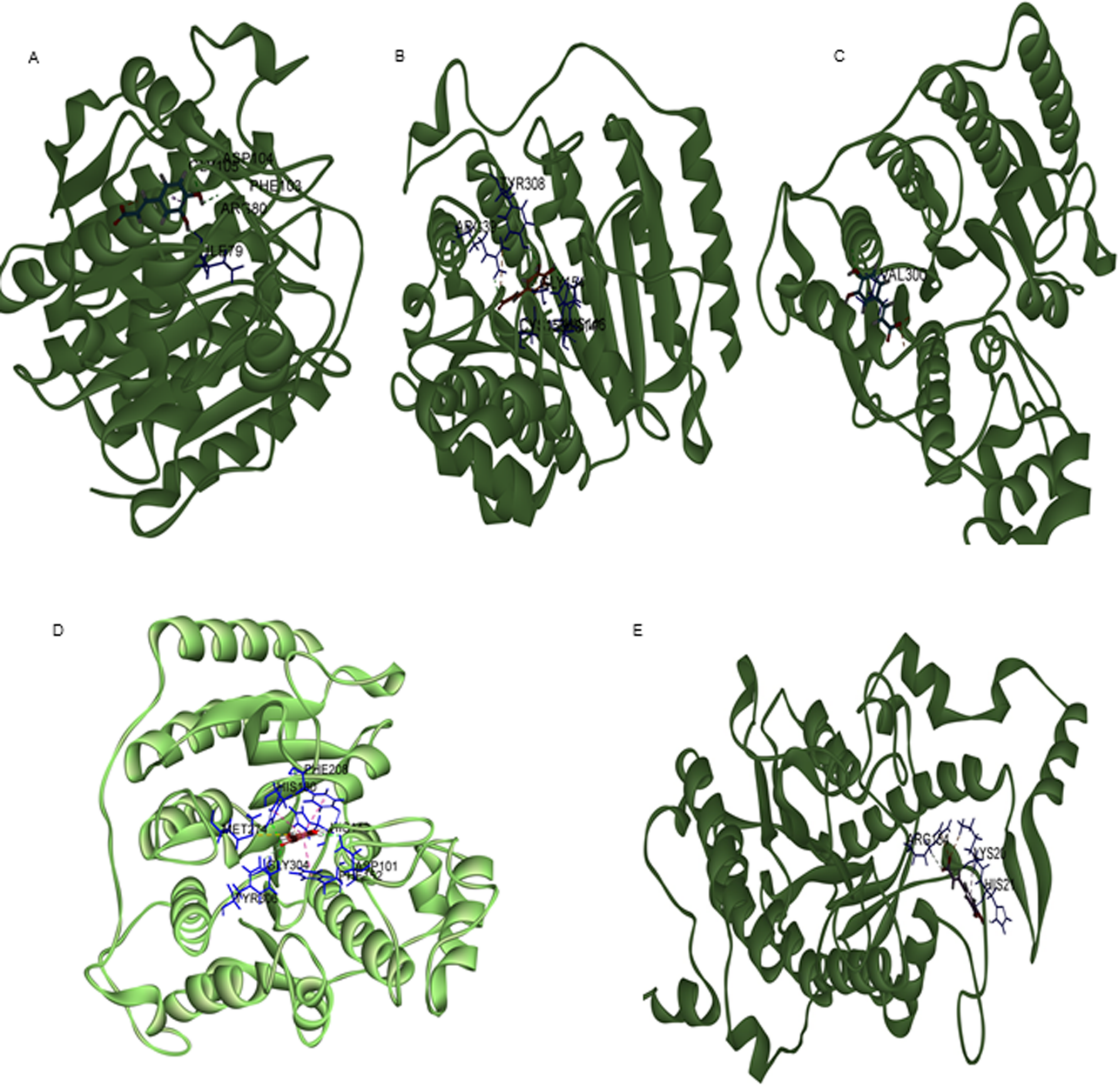
(A) DHCA interacted with HDAC1 by forming three hydrogen bonds with I79, F103 and G105 amino acid residues (B) The key amino acids residues of HDAC2 such as H145, H146, G154 and Y308 were involved in hydrogen bonding with DHCA (C) While interaction between HDAC3, and DHCA was by formation of hydrogen bond with—R301 (D) The amino acids D101, H180 and Y306 of HDAC8 interacted with DHCA. (E) DHCA also interacted with class II HDAC-4 by forming bonds with ARG154, LYS20 and HIS21.

Class-I HDACs consisting of HDAC1, HDAC2, HDAC3 and HDAC8, are well known oncogenic proteins expressed at high levels in malignant cervical and colorectal cancers [41]. Among all the HDACs of class 1, HDAC2 is overexpressed in colon cancer tissues (81.9%) compared to normal colon tissue (53.1%) [5,42]. Since prior studies have demonstrated the anti-cancer potential of pharmacological agents targeting HDACs, an attempt was made to screen and identify key cinnamic acids for inhibiting HDAC thereby the cancer cell proliferation[43]. The binding efficacy of CA, hydroxy CAs (HCA and DHCA) and methoxy derivatives of CA (MHMMCA, DMCA, MHDMCA, TMCA) for docking in to TSA binding site of HDAC isoforms was determined using Discovery Studio software as described in methods section. Experimentally, first, the X-ray crystal structure of Class-I (HDAC-1,2,3 and 8) and class-II HDACs (HDAC-4) was retrieved from protein data bank and docked with CA derivatives (Fig 1A, 1B, 1C, 1D and 1E) and known HDAC inhibitor Trichostatin A (S1A, S1B, S1C, S1D and S1E Fig) and. Next, the C-docker energy and molecular interactions were calculated for TSA, CA and CA derivatives to select a potent CA derivative for inhibiting HDACs (Table 2). Among CA derivatives tested, DHCA exhibited stronger interactions with all HDAC isoforms as evidenced by lower C-docker energy compared to even the positive control TSA. DHCA exhibited highest binding to HDAC2 and HDAC3 of class I. Subsequent analysis assessing the molecular interaction of DHCA, especially the hydrogen bonds, with different isoforms of HDAC demonstrated the ability of DHCA to interact strongly with Class-1 HDACs—HDAC1, HDAC2, HDAC3 and HDAC8 through hydrogen bond formation. Detailed analysis showed that DHCA formed three hydrogen bonds by interacting with I79, F103 and G105 amino acids of HDAC1 (Fig 1A). Similarly, DHCA is involved in forming hydrogen bonds with key amino acids of HDAC2—H145, H146, G154 and Y308 (Fig 1B); HDAC3—R301(Fig 1C) and with HDAC8 D101, H180 and Y306(Fig 1D). With Class II HDAC-4, DHCA interacted with higher c-docker energy forming bonds with ARG154, LYS20 and HIS2. In addition, the HDAC2 and HDAC3 structures were screened for SAHA and Sodium butyrate binding sites and the determined sites were docked with DHCA to understand the extent of interaction. Sodium butyrate and SAHA interacted on multiple sites with HDAC2 and HDAC3. Only the sites where both DHCA and the reference compounds (SAHA and Sodium butyrate) interacted were considered for the comparison of binding potentials. Among SAHA and DHCA, SAHA (-44.23kcal/mol) demonstrated better interaction with HDAC2 when compared to DHCA (-36.66kcal/mol). However DHCA (-37.297 kcal/mol) demonstrated stronger binding to HDAC 3 when compared to SAHA (-31.661kcal/mol)(S2A, S2B, S2C and S2D Fig). DHCA (-36.66 kcal/mol for HDAC 2 & 37.078 kcal/mol for HDAC3) demonstrated stronger binding to both HDAC 2 and HDAC3 when compared the binding potential to Sodium butyrate (-31.7202 kcal/mol for HDAC1 & 35.7014 kcal/mol for HDAC2) (S3A, S3B, S3C and S3D Fig)

**Table 2:** Docking energy and c-docker interactions of CA derivatives with HDAC at TSA binding site.

S.No	Molecule Name	Pubchem CID	C Docker Energy (Kcal/Mol)					
			HDAC1	HDAC2	HDAC3	HDAC8	HDAC4	
1	CA	444539	-23.1768	-28.2345	-29.3325	-19.7442	-25.8947	
2	MHMMCA	445858	-26.036	-30.6062	-36.5255	-19.6301	-32.4985	
3	MHCA	637542	-25.6455	-30.9827	-34.2941	-24.7345	-32.6188	
4	DHCA	689043	-31.5175	-36.66	-37.297	-27.5259	-38.4799	
5	DMCA	717531	-21.9594	-24.4914	-31.5091	-17.4747	-26.7209	
6	TMCA	735755	-14.6236	-8.75977	-25.3784	-9.63857	-11.8894	
7	MHDMCA	10743	-23.2463	-12.8865	-32.3465	-16.0152	-23.1942	
8	Trichostatin-A	444732	-9.78099	-14.8177	-8.35061	-10.2706	-1.32476	

The strong binding of DHCA with different isoforms of HDAC at different binding sites may be due to the presence of hydroxyl groups, which has the ability to strongly interact with the amino acid residues of the enzymes in the active site as compared to the methoxy derivatives.

Table 2 describes the docking energy of CA derivatives and TSA with different isoforms of Class I HDAC (HDAC1,2,3 and 8) and class II HDAC (HDAC-4) enzyme. Among the CA derivatives, DHCA showed much lesser docker energy compared to other CA derivatives and TSA.

Table 3 describes the c-docker energy of DHCA, Sodium butyrate and SAHA with HDAC2 and HDAC3.

**Table 3:** Docking energy and c-docker interactions of DHCA at sodium butyrate (Pubchem CID 5222465) and SAHA (Pubchem CID 5311) with HDAC2 and HDAC3.

Name of the compound	C Docker Energy (Kcal/Mol)	
	HDAC2	HDAC3
SAHA BINDING SITE		
SAHA	-44.236	-31.661
DHCA	-36.660	-37.297
SODIUM BUTYRATE SITE		
Sodium butyrate	-31.720	-35.701
DHCA	-36.660	-37.078

### DHCA and sodium butyrate inhibited the HDAC activity in ex vivo biochemical assay

**Fig 2:**
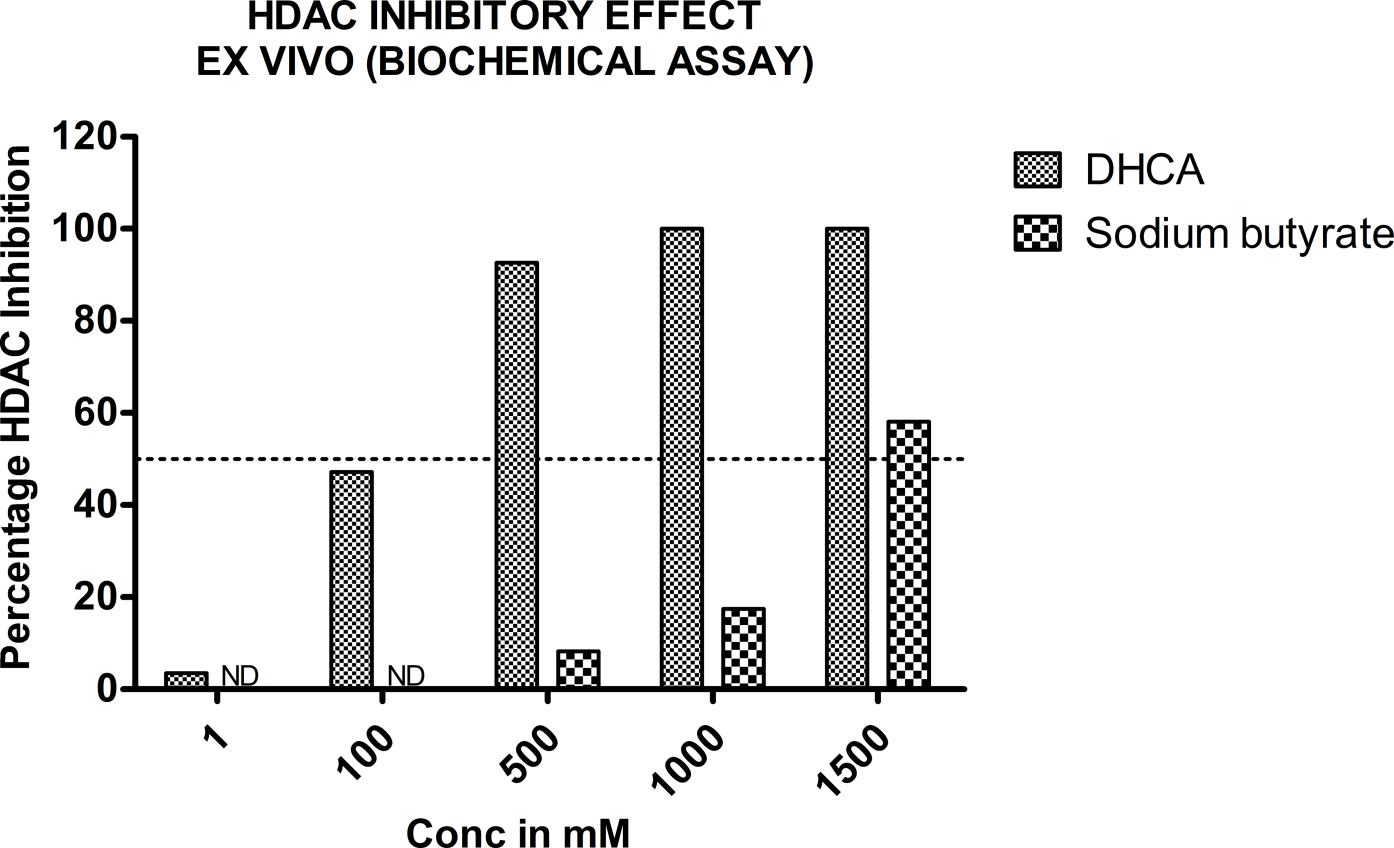
DHCA and sodium butyrate inhibited HDAC activity ex vivo: To assess whether DHCA (which showed better HDAC inhibition in silico) inhibits HDAC activity ex vivo, HDAC enzyme (isolated from HeLa cells-provided in kit) was incubated with increasing concentrations of DHCA for 20 minutes and the percentage inhibition calculated. Analysis of the data demonstrated much better HDAC inhibition with DHCA compared to sodium butyrate (a well-known HDAC inhibitor). TSA, another known HDAC inhibitor used as positive control in this study, showed much stronger HDAC inhibition compared to DHCA or sodium butyrate.

In order to confirm the in silico data, which identified DHCA as a potent HDAC inhibitor, an ex vivo study assessing the ability of DHCA to decrease HDAC enzyme (extracted from HeLa cell nuclear extract) activity was carried out. In brief, the nuclear extract of HeLa cells was incubated with increasing concentrations of DHCA and sodium butyrate (a known HDAC inhibitor) and percentage inhibition compared to untreated controls calculated. An about 47% and 97.67% activity of HDAC was inhibited, respectively by 100μM and 500μM of DHCA (Fig 2A). Sodium butyrate did not show any inhibitory effects up to 500μM. However at 1000μM and 1500μM an about 17% and 58% HDAC inhibition was observed (Fig 2A). Trichostatin A was used as a positive control which showed 97.3% inhibition at 1.0μM.

### DHCA inhibited HDAC activity ex vivo as well as in vitro in colon cancer cell lines

Since colon cancer tissues are known to express high HDAC activity compared to normal colon tissues, the HDAC inhibitory effect of DHCA was assessed using colon cancer cells both by ex vivo (biochemical assay) and in vitro (cell based assay) methods.

**Fig 3:**
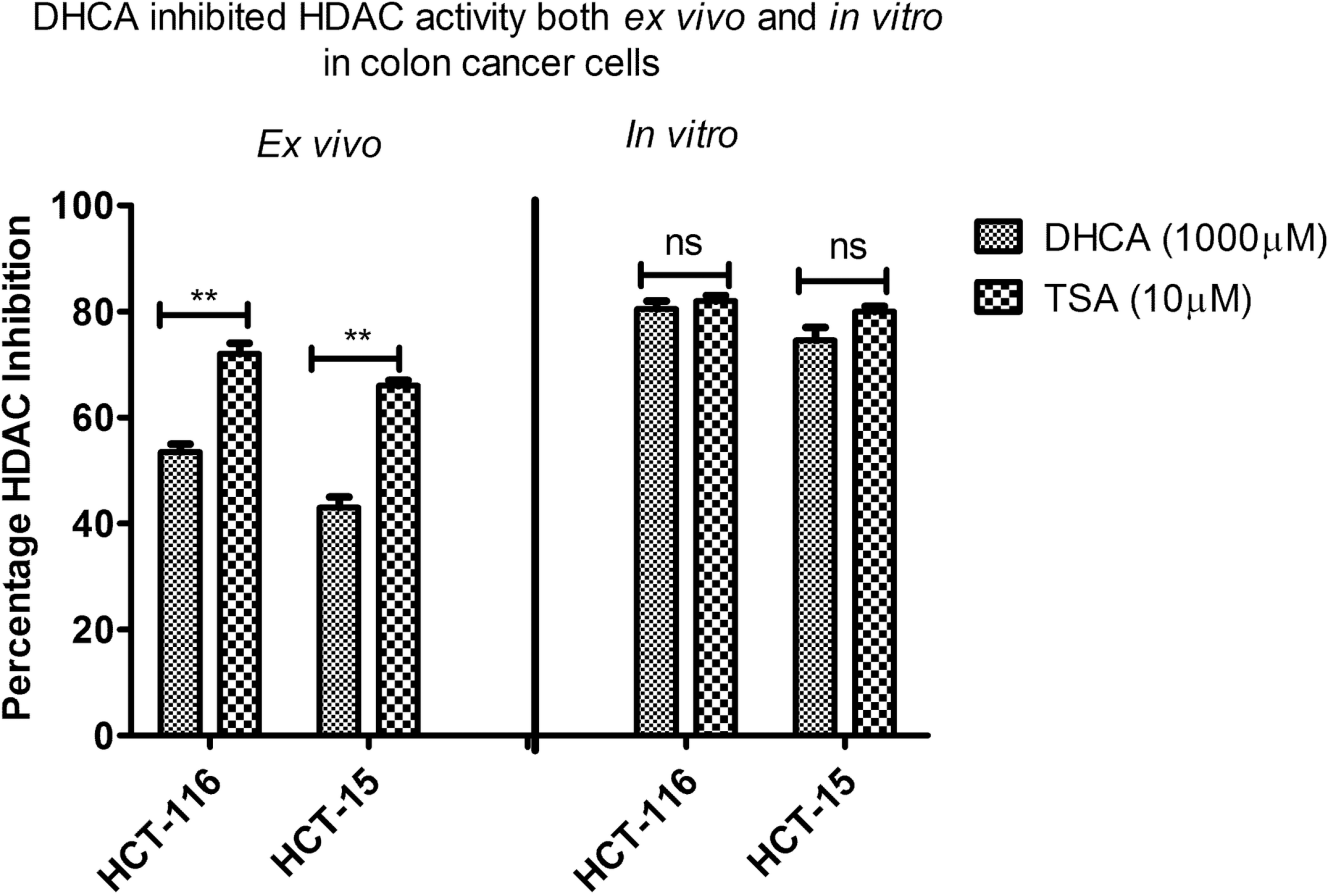
DHCA inhibited the activity of HDAC ex vivo and in vitro: DHCA effectively inhibited HDAC activity both ex vivo and in vitro at 1000μM. Ex vivo treatment of nuclear extracts of HCT-116 and HCT-15 cells exhibited 52% and 43% HDAC inhibition, respectively in HCT-116 and HCT-15 cell lines. However, treating the colon cancer cells with DHCA for 48h showed much better HDAC inhibitory effect (80% and 77% HDAC inhibition, respectively in HCT116 and HCT15 cell lines).

First, the nuclear extracts of colon cancer cell lines (HCT-116 and HCT-15) were treated with 1000μM of DHCA and effect on HDAC inhibition analysed using biochemical assay. DHCA inhibited the activity of HDAC collected from HCT-116 and HCT-15 cell lines by 53% and 43%, respectively. Similar to other assays, TSA showed much better inhibition (80% and 77%, respectively, in HCT-116 and HCT-15 cell lines (Fig 3).

Next, to determine whether addition of DHCA to exponentially growing cell lines also exhibited similar inhibition pattern, HCT116 and HCT15 cells were treated with DHCA at 1000μM for 48h and nuclear extracts collected as detailed in materials and methods. The data demonstrated that DHCA could reduce HDAC activity by about 80% and 77% respectively in HCT116 and HCT15 cells compared to vehicle DMSO treated cells (Fig 3). Trichostatin-A, showed 80% and 81% HDAC inhibitory effect, respectively, on HCT-116 and HCT-15 cell lines when exposed for 48h (Fig 3).

### HDAC inhibitor DHCA retarded the growth of cell lines representing carcinomas of cervix, and colon and rectum

**Fig 4:**
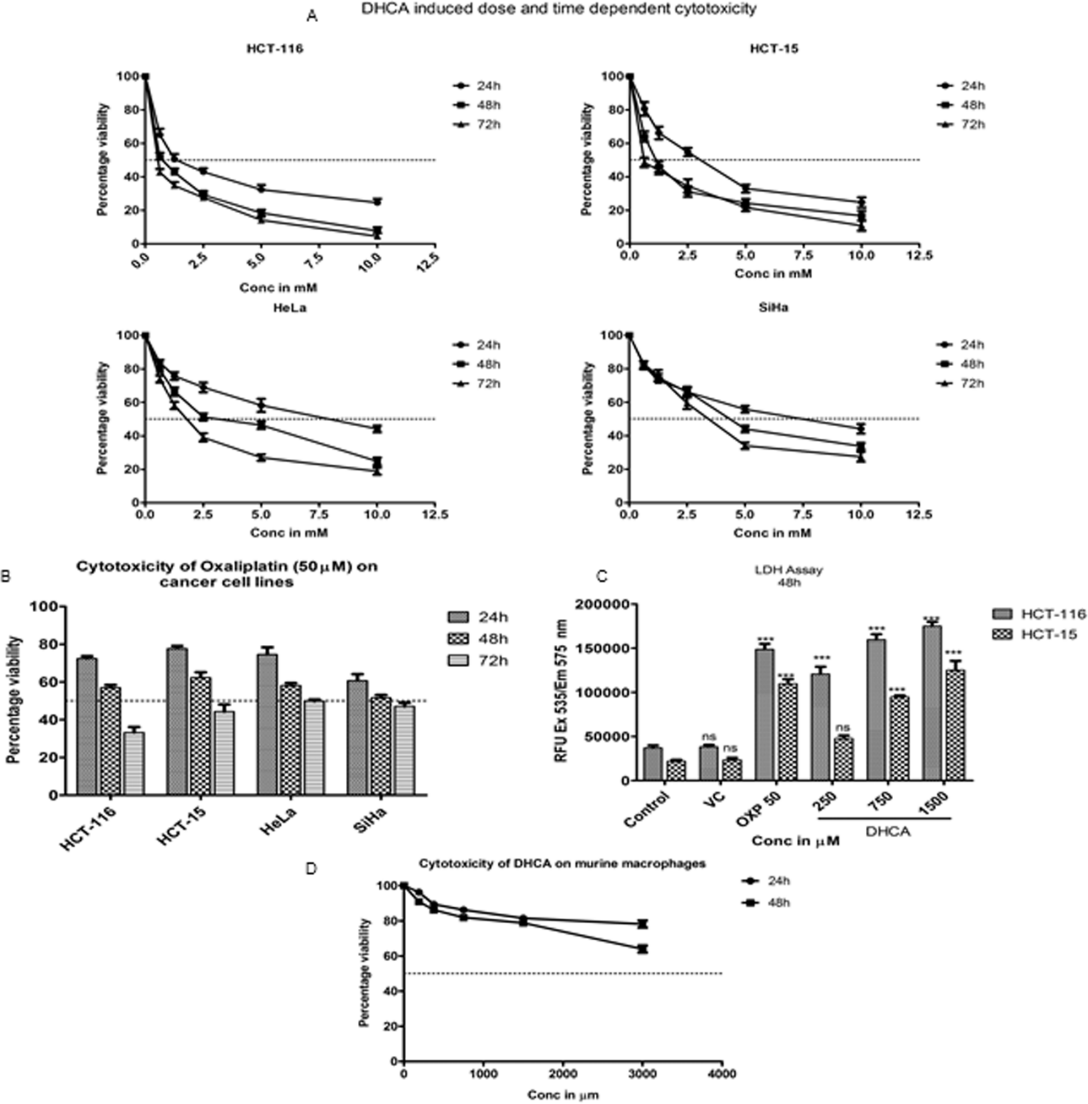
DHCA reduced cell viability and induced apoptosis in cancer cell lines: (A) DHCA inhibited the growth of colorectal carcinoma cell lines HCT-116 and HCT-15 and cervical carcinoma cell lines HeLa and SiHa in a time and dose dependent manner. (B) 50μM Oxaliplatin induced cancer cell death in colon and cervical cancer cells at 24h, 48h and 72h. (C) Induction of cancer cell death by DHCA treatment enhanced the release of lactate dehydrogenase from cells at 48h. VC indicates vehicle control (1% DMSO). (D) DHCA did not inhibit the viability of Raw 264.7 (mouse macrophage cell line) cells even at the dose of 3000μM.

Although DHCA exhibited potent inhibitory activity against HDAC enzyme present in cervical and colon cancer cell lines, its effects on retarding the growth of these cell lines is not known. Therefore a dose and time dependent cytotoxicity assay was performed on cell lines representing cervical (HeLa and SiHa) and colorectal (HCT-116 and HCT-15) cancers (Fig 4A). The data demonstrated a time- and dose- dependent cell growth inhibition with colorectal carcinoma cells exhibiting more sensitivity to compound treatment compared to cervical cancer cells (Fig 4A). For example, the IC_50_ values for colorectal carcinoma cells HCT116 and HCT15 were 0.5mM and 0.704mM, respectively, compared to HeLa and SiHa, for which the IC_50_ values were 1.66mM and 3.22mM at 72h of treatment (Table 4). 50μM Oxaliplatin was used as a positive control for cytotoxic effect (Fig 4B). The cytotoxic effect of DHCA was further confirmed by the results of lactate dehydrogenase assay. Results of LDH assay (Fig 4C) showed an about 4.5–5 fold increase in LDH activity upon the treatment of HCT-116 and HCT-15 cells with DHCA (Fig 4C). Next, to determine the selectivity of DHCA, Raw 264.7 (a mouse macrophage cell line) cells were exposed to increasing concentration of DHCA and the number of viable cells estimated after 48h treatment. The data depicted in Fig 3D showed no significant cell death upon exposure of RAW 264.7 cells to DHCA indicating that these compounds are selective to cancer cells (Fig 4D).

**Table 4:** IC_50_ values (in mM) of DHCA on colon and cervical cancer cell lines.

Cell line	24h		48h		72h	
	Mean	SE	Mean	SE	Mean	SE
HCT-116	1.734	0.218	0.742	0.016	0.500	0.013
HCT-15	2.595	0.143	1.188	0.068	0.704	0.010
HeLa	8.148	0.192	3.018	0.061	1.66	0.088
SiHa	6.942	0.088	4.511	0.098	3.223	0.095

Table 4 describes the IC_50_ values of DHCA on colon and cervical cancer cell lines at 24h, 48h and 72h. The IC_50_ values calculated using GraphPad Prism version 5.0 indicate that colon cancer cell lines were more susceptible to DHCA treatment compared to cervical cancer cell lines.

### DHCA elevated reactive oxygen species and arrested cells in G2/M phase of the cell cycle

**Fig 5:**
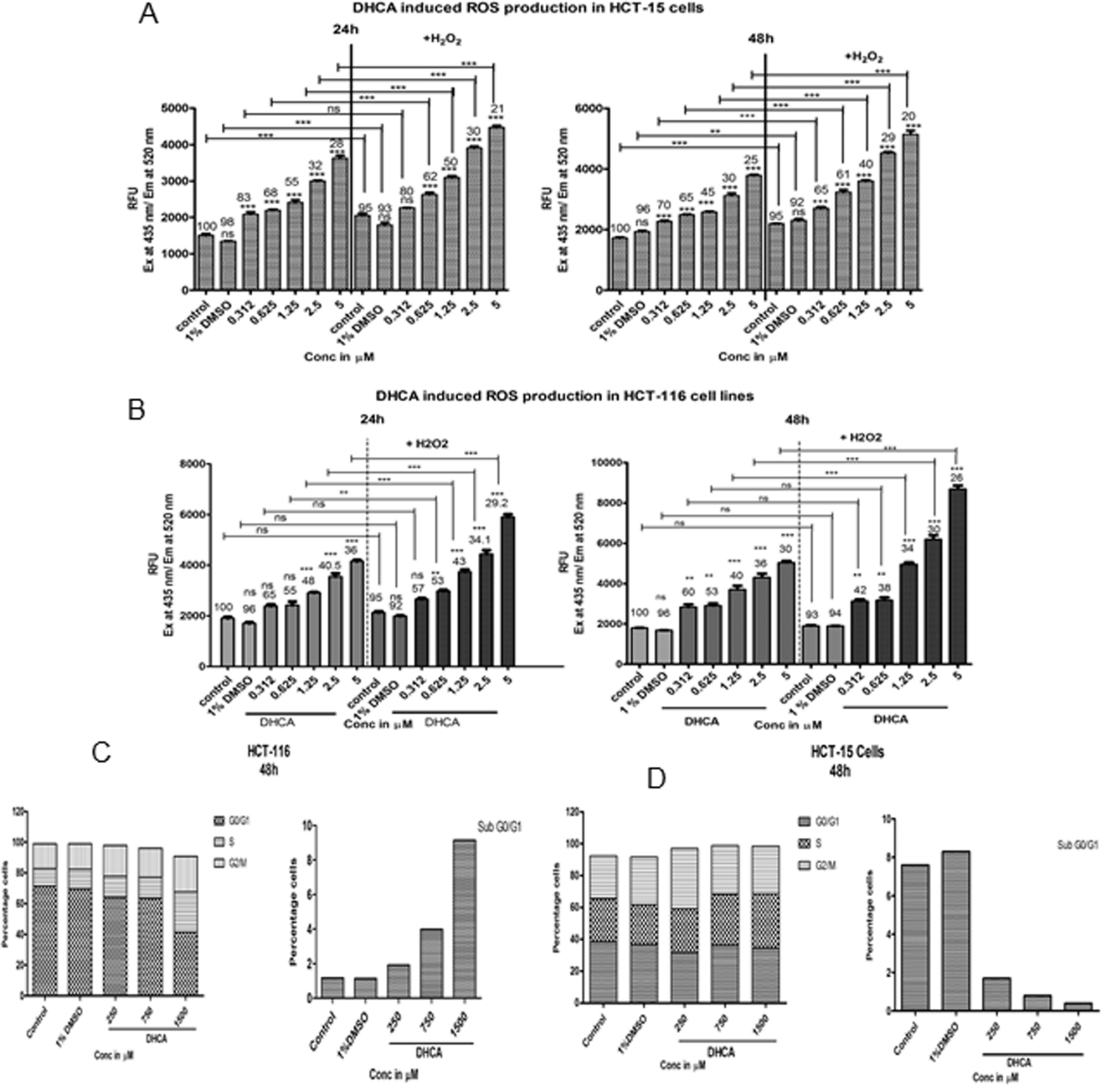
DHCA induced ROS generation and arrested cells in S- and G2/M phase of cell cycle: (A) HDAC inhibitor DHCA increased ROS levels in a dose and time dependent manner to induce cell death in HCT-116 cell lines. Further, in presence of an external ROS generator like H_2_O_2_, the production of ROS was much higher compared to H_2_O_2_ and DHCA alone, indicating that these molecules cause cell death through ROS induction. The numbers above the graph indicate percentage viable cells. (B) DHCA also enhanced the ROS production in HCT-15 cell lines similar to HCT-116 cells. The effect of compounds on ROS production was found to be dose dependent. (C) Analysis of HCT-116 cells treated with DHCA showed induction of apoptotic sub-G0-G1 population and arrest of cells in S- and G2/M phase. (D) DHCA treatment arrested HCT-15 cells in S phase in a dose dependent fashion.

Since several prior studies have shown induction of reactive oxygen species upon HDAC inhibition, the levels of ROS were quantitated in cells exposed to or not exposed to DHCA. Treatment of HCT-116 (Fig 5A) and HCT-15 (Fig 5B) with DHCA showed a significant increase in reactive oxygen species at 24h and 48h. Furthermore, addition of H2O2 to DHCA treated cells further elevated ROS levels significantly thereby enhanced cellular damage (Fig 5A and 5B). Elevated ROS causes cell cycle arrest by damaging cellular proteins and lipids. Therefore, pharmacological agents that can induce ROS and interrupt the cell cycle progression are potential anti-cancer agents. Treatment of HCT116 and HCT-15 cells with DHCA for 48h induced apoptosis as evident by accumulation of cells in Sub G0/G1 phase, and arrest of cells in S and G2/M phases (Fig 5C and Fig 5D) (S4A and S4B Fig) [44,45]. Oxaliplatin, a positive control used in this study, arrested cells in G2-M phase.

### DHCA induced the expression of p21 in cell lines representing colorectal carcinomas

**Fig 6:**
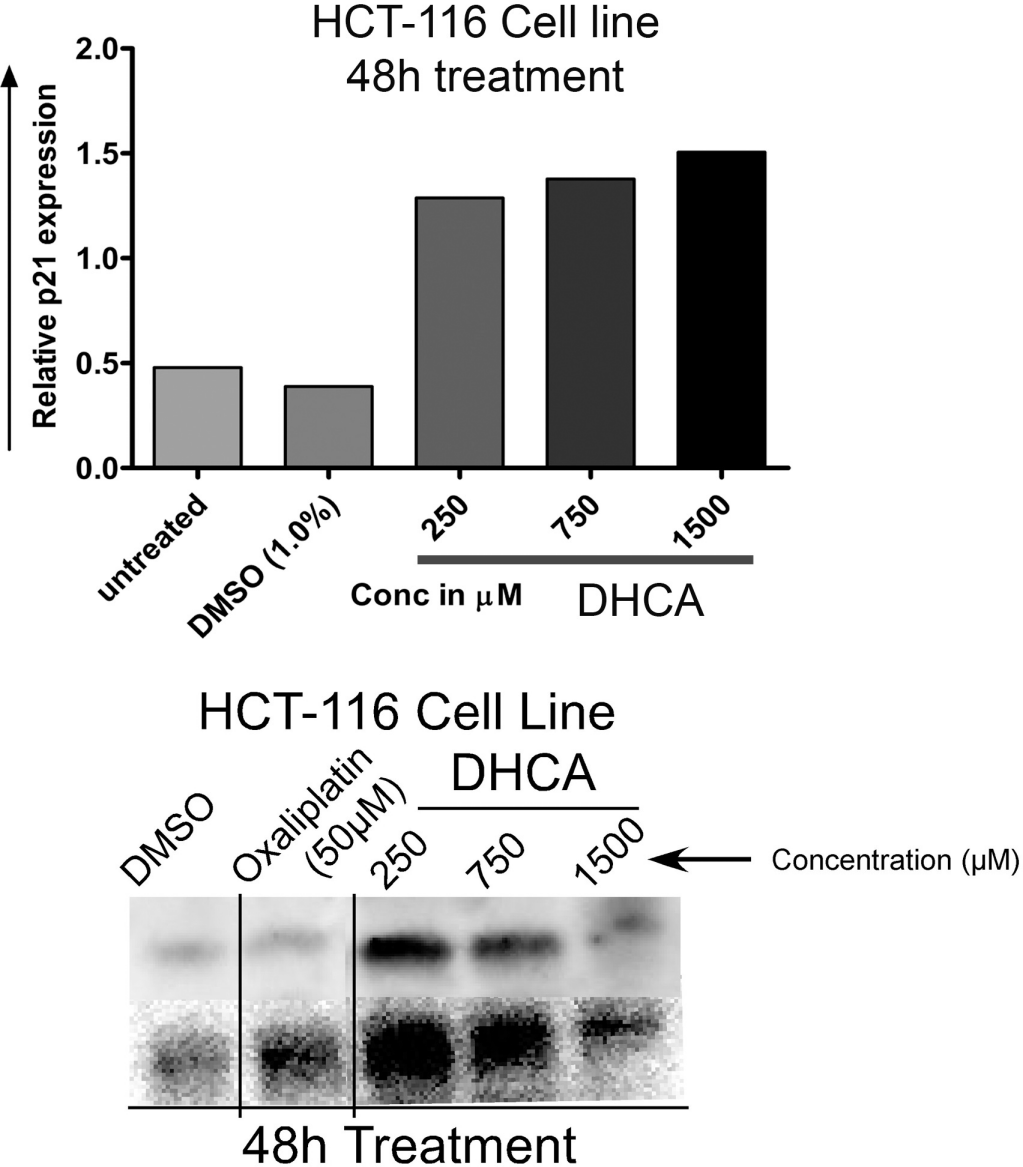
100μg of protein collected from DHCA treated (250, 750 and 1500μM) and untreated HCT-116 cells (48h) were subjected to western blot for estimation of p21 expression. The data revealed that DHCA induced the expression of tumor suppressor gene p21 and thus could inhibit the proliferation of cancer cells. The protein bands were quantified using image-J software and fold increase in expression plotted.

In order to check whether HDAC inhibitor DHCA induce the expression of p21, protein lysates from HCT-116 cell lines exposed to DHCA for 48h were subjected to immunoblotting as described in methods section. An about 2.8 and 3.1 fold increase, compared to untreated cells, in the expression of p21 was obsereved when HCT116 cells were treated with 750μM and 1500μM DHCA for 48h (Fig 6). Therefore like many other HDAC inhibitors DHCA also induced the expression of p21 and thereby inhibited the cell cycle kinases to control cellular proliferation [46].

### DHCA induced apoptosis in colon cancer cells by triggering caspase-3 levels

**Fig 7:**
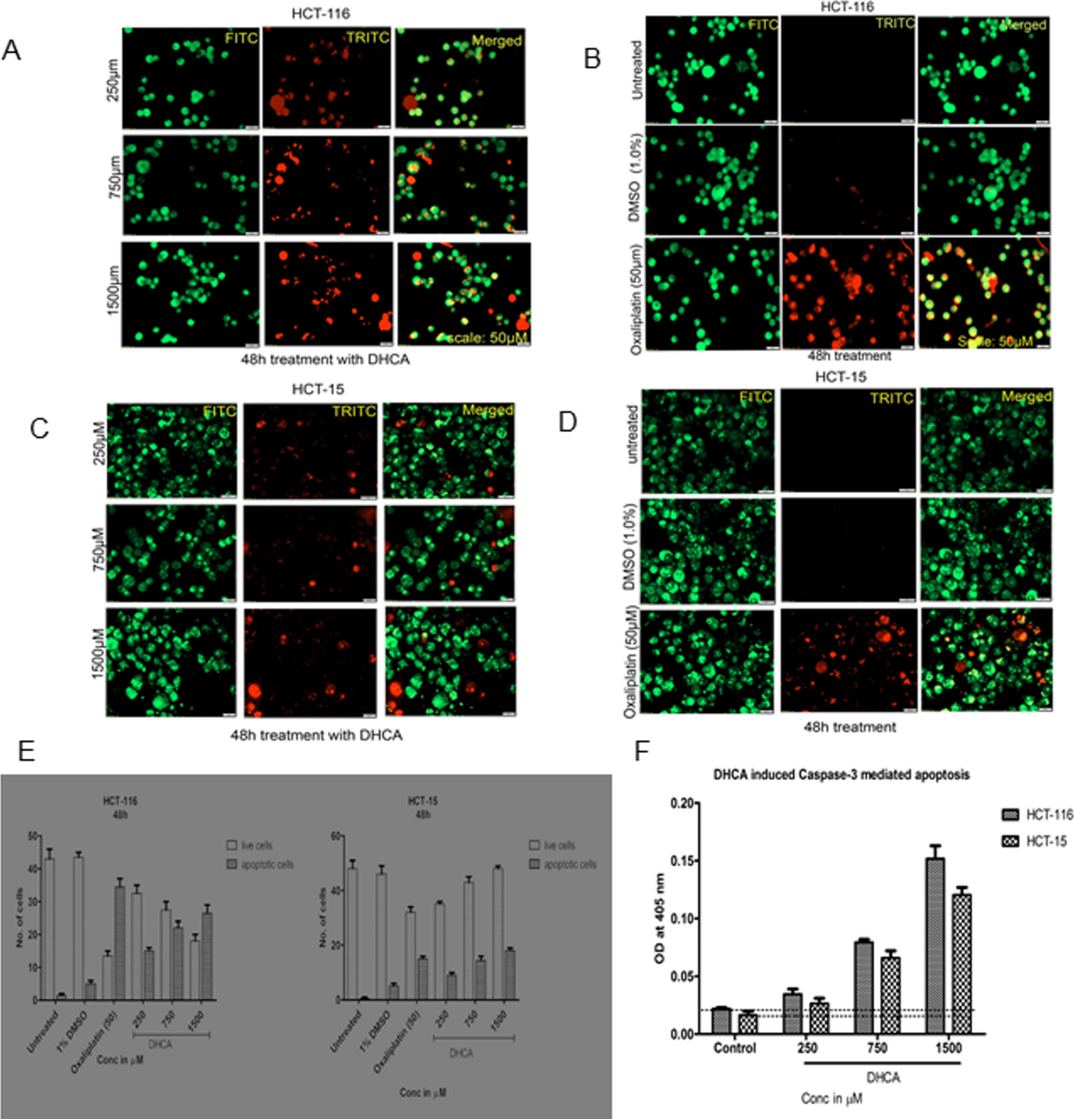
DHCA induces apoptosis in colon cancer cell lines: (A) Acridine orange and ethidium bromide staining of HCT-116 cancer cells indicated that DHCA induced apoptosis as evidenced by the presence of condensed chromatin (orange stained cells) (B) Untreated HCT-116 cells appear green indicating no apoptosis. Images were captured using FITC and TRITC at 10X magnification. The captured images were later merged. (C) Increased appearance of orange stained HCT-15 cells indicates the induction of apoptosis by DHCA at 48h. (D) Untreated and vehicle treated (1% DMSO) HCT-15 cells appear green while the oxaliplatin treated cells appeared orange. (E) The number of live and apoptotic cells were quantified and plotted. With increasing concentrations of DHCA increase in apoptotic (orange cells) cells also increased at 48h. (F) DHCA treated colon cancer cells expressed high levels of caspase-3, an apoptotic marker, compared to vehicle treated cells, confirming the induction of apoptosis.

In order to further check whether DHCA treatment induced apoptosis in colorectal cancer cell lines, first, the DHCA exposed and un-exposed cells were stained with acridine orange and ethidium bromide; and next measured the levels of caspase-3 (Fig 7). In brief, HCT-116 (Fig 7A and 7B) and HCT-15 (Fig 7C and 7D) cell lines were treated with increasing concentrations of DHCA for 48h and stained with ethidium bromide and acridine orange. Acridine orange stains the nucleus green by penetrating into all cells (live and dead) while ethidium bromide enters cells only when cytoplasmic integrity is lost and stains the nucleus red. Thus the apoptotic cells where membrane integrity is lost appear orange (Fig 7A and 7C) while normal cells appear green (Fig 7B and 7D). Analysis of photomicrographs showed significantly high orange-stained cells when treated with DHCA which were absent in untreated or vehicle 1% DMSO treated cells. The number of orange stained cells increased with increasing DHCA concentration (Fig 7E).

Corroborating with acridine orange and ethidium bromide staining data, the expression of caspase-3 was also elevated in the DHCA treated cells compared to untreated and 1% DMSO vehicle treated cells (Fig 7F). Analysis of the data demonstrated a dose dependent increase in caspase-3 expression when HCT-15 and HCT-116 cells were treated with increasing concentration of DHCA (Fig 7F). For example, a significant 3- and 7- fold high caspase-3 compared to DMSO treated cells was observed, respectively, when HCT-15 and HCT-116 cells were exposed to 1500μM DHCA (Fig 7F). In total, the data showed that DHCA induced apoptosis was mediated by caspase-3 expression in colorectal carcinoma cell lines.

## Discussion

Aberrant changes in gene expression controlled by HDAC/HAT enzymes can transform normal cells in to cancerous ones and induce resistance to chemotherapeutic agents [47]. Therefore proper control over the expression and activity HDAC/HAT is much essential to prevent and treat tumors [48]. Unusually high HDACs can modulate the expression of genes by epigenetic mechanisms to drive the formation of tumors. Elevated expression and activity of HDAC has been reported in several cancers [49]. HDACs, by removing the acetyl groups from histones, can create a non-permissive chromatin conformation, which prevents the transcription of genes involved in (a) proliferation inhibition such as cyclin dependent kinase inhibitors (CDKIs); (b) the expression of pro-apoptotic proteins; and (c) differentiation [47]. For example, HDACs prevents the transcription of tumor suppressor proteins p21 and PTEN [50]. In addition to histones, HDACs can deacetylate a variety of other proteins that include (a) transcription factors such as STAT3 and P53; (b) retinoblastoma proteins; and (c) proteins implicated in control of cell growth (FOXO1), differentiation and apoptosis. Therefore targeting HDACs could be a promising therapy to inhibit cancer cells. Although several pre-clinical and early clinical studies have tested the safety and efficacy of HDAC inhibitors such as SAHA, the success of these drugs as monotherapy for treating cancers is minimal [19,51]. Hence, identification of new HDAC inhibitors with minimal side effects is warranted. Recently several HDAC inhibitors have been developed and tested against carcinomas of skin (Entinostat) and prostate (Practinostat). However, these inhibitors are still in the clinical development (Phase-II trials completed) and require Phase-III and IV testing. Therefore, the search for safe and potent HDAC inhibitors still continues.

Phenolic compounds that include derivatives of benzoic acid and cinnamic acid are well known HDAC inhibitors and anticancer agents with minimal side effects [24]. A recent study from our group demonstrated that benzoic acids with increasing number of hydroxylic groups could inhibit HDAC activity more effectively compared to benzoic acid with no hydroxylic group substitution [20]. However, it is not known, whether similar trend (ie., increased potency with increase in hydroxylic group substitution) is also observed with cinnamic acids. Hence, we have tested the efficacy of cinnamic acids, with varied number of hydroxylic groups, for inhibiting HDAC activity by docking the CA-derivatives in to TSA binding site and validated the in silico data with ex vivo and in vitro assays. The molecular docking and ex vivo HDAC inhibitory activity assay methods have identified dihydroxy cinnamic acid (DHCA) as the potent down-modulator of HDAC. DHCA bound to TSA binding site of HDAC thereby inhibited its activity. Several studies using pharmacological agents targeting HDAC activity or siRNA-mediated knock down of the expression of HDAC have shown reduced cell proliferation indicating tumor growth retarding ability of HDAC inhibitor [52–54]. Treatment of cell lines representing carcinomas of cervix and colon and rectum with DHCA inhibited HDAC activity and reduced cell proliferation in vitro in a dose dependent manner demonstrating the ability of naturally occurring cinnamic acid derivatives for retarding the growth of cancer cells. Among the HCT-116 and HCT-15 cell lines used for the study, HCT-116 cell line was found to be sensitive to DHCA compared to HCT-15 which could be due to the modifications of the early immediate genes. Differential sensitivity of HDAci on cell lines is been previously reported by Wilson AJ et al 2010 and Chueh et al 2017[55,56].

HDAC inhibitors are known to arrest cells in cell cycle [57]. For example, SAHA retarded cancer cell growth in S and G2/M phases [58]. Exposing cells to DHCA elevated sub-G0-G1 population (an indicator of apoptosis) and arrested cells in S- and G2/M phases [59]. Recent studies by Du L et al., 2014 and Karthik et al., 2014 showed G1/S or G2/M phase arrest when cancer cells were treated with HDAC inhibitors such as romidepsin and vorinostatin [44,45].

The cell cycle arrest induced by HDAC inhibitors is in part mediated by the upregulation of p21, a known suppressor of cell division [60]. Data from our studies also demonstrated similar trend in the p21 upregulation when colorectal cancer cells were exposed to DHCA, further indicating that the cell growth inhibition triggered by DHCA is mediated by the inhibition of HDAC. In addition to cell proliferation inhibition, targeted reduction of HDAC activity using small interfering RNA or pharmacological agents promotes apoptosis in cancer cells [61]. The induction of apoptosis is mediated by the activation of caspases as well as by promoting the cellular reactive oxygen species [6,62–64]. Data presented in this study also demonstrated that HDAC inhibition using DHCA significantly increased tumor cell apoptosis through the upregulation of active caspase as well as by enhancing ROS. Therefore, the HDAC inhibitor DHCA is a potent inducer of cancer cell death.

## Conclusion

In summary, DHCA showed potent HDAC inhibition as evidenced by its ability to dock in to TSA binding site of HDAC. Inhibition of HDAC activity using DHCA triggered apoptosis in cancer cells, arrested the progression of cells through G2/M, and elevated the expression of ROS, p21 and caspase-3 levels. Hence, DHCA could be a potent HDAC inhibitor for considering it for further development.

## Supporting information

S1 Fig In silico analysis revealed that TSA binds to HDAC1, HDAC2, HDAC3, HDAC8 of class I and HDAC4 of class II. (A): Trichostatin A, a well-known inhibitor of HDAC binds to HDAC 1 by interacting with key amino acid residues such as LEU76, ILE79, ARG80, ASP82, ASP104 and PHE103. (B) TSA had better interactions with HDAC2 compared to other HDAC of Class 1 by interacting with HIS183, HIS146, LEU144, ASP104, CYS156 and MET35. (C) Interaction between TSA and HDAC3 was by formation of bonds with aminoacid residues like HIS17, TYR331, VAL300, and ARG301. (D) TSA interacted with HDAC8 by linking with PHE152, GLY151, PHE208, HIS180 and TYR306. (E) TSA interacted with Class II HDAC-4 by forming bonds with HIS54 residue.
(TIF)

S2 Fig DHCA interacted with HDAC2 and HDAC3 at SAHA binding sites. (A): HDAC inhibitor SAHA interacted with HDAC2 with key amino acid residues such as ALA141, HIS 146, HIS183, ASP269, PHE155, PHE210 (B) DHCA bound to SAHA binding site at HDAC2 by interacting with HIS145, HIS146, TYR308, GLY154, CYS156 (C) SAHA interacted with ARG301 and CYS263 of HDAC3 (D): DHCA also interacted with HDAC3 at ARG301, ARG265 and LYS25.
(TIF)

S3 Fig DHCA interacted with HDAC2 and HDAC3 at Sodium butyrate binding sites. (A): Sodium butyrate a known HDAC inhibitor interacted with HDAC2 with key amino acid residues such as HIS145, HIS183 and TYR 308 (B) DHCA interacted with HDAC2 at sodium butyrate binding site by forming bonds with HIS145, HIS146, TYR308, GLY154, CYS156 (C) Sodium butyrate interacted with ARG265 and LYS25 of HDAC3 (D): DHCA also interacted with HDAC3 at ARG301, ARG265 and LYS25.
(TIF)

S4 Fig Exposure of colon cancer cells to DHCA for 48h arrested cells at G2/M phase. (A): DHCA arrested HCT-116 cell lines in G2/M phase ion a dose dependent manner when compared to untreated and vehicle treated cells at 48h. (B) DHCA also inhibited HCT-15 cells in a dose dependent fashion at 48h.
(TIF)

## Abbreviations

CA: cinnamic acid
BCA: Bicinchoninic acid
BSA: Bovine Serum Albumin
DHCA: Dihydroxy cinnamic acid
DMCA: Dimethoxy methoxy cinnamic acid
DMEM: Dulbecco’s Modified Eagle Medium
DMSO: Dimethyl sulfoxide
EDTA: Ethylene diamine tetra acetic acid
EGTA: Ethylene glycol-bis(2-aminoethylether)- tetra acetic acid
FBS: Fetal bovine serum
HAT: Histone acetyl transferase
HDAC: Histone deacetylase
HEPES: 4-(2-hydroxyethyl)-1-piperazineethanesulfonic acid
LDH: Lactate dehydrogenase
MHDMCA: Monohydroxy dimethoxy cinnamic acid
MHMMCA: Monohydroxy monomethoxy cinnamic acid
MgCl_2_: Magnesium chloride
NaCl: Sodium chloride
NP- 40: Nonidet P-40
PDB: Protein data bank
PI: Propidium iodide
RCSB: Research collaboratory for structural bioinformatics
RIPA buffer: Raidoimmunoprecipitation Assay buffer
ROS: Reactive oxygen species
SAHA: Suberanilohydroxamic acid
SRB: Sulforhodamine b
TBST: Tris buffered Saline with tween
TCA: Trichloro acetic acid
TMCA: Trimethoxy cinnamic acid
TSA: Trichostatin A
