# Supplementary figures and images for: Induction of colon and cervical cancer cell death by cinnamic acid derivatives is mediated through the inhibition of Histone Deacetylases (HDAC)
Source: PLoS One. 2017 Nov 30;12(11):e0186208. doi: 10.1371/journal.pone.0186208 (PMC5708809; doi:10.1371/journal.pone.0186208)

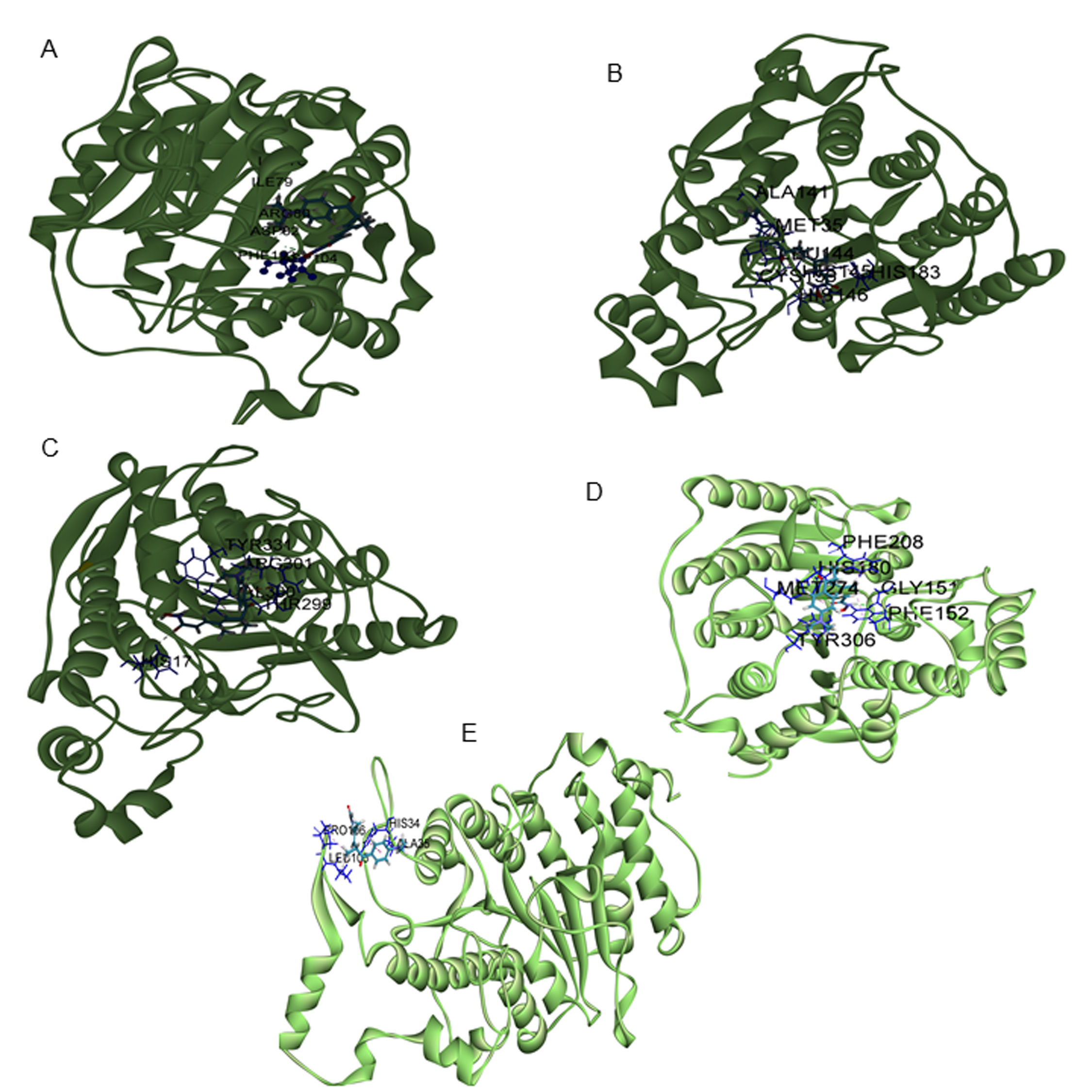

Supplement: S1 Fig — (A): Trichostatin A, a well-known inhibitor of HDAC binds to HDAC 1 by interacting with key amino acid residues such as LEU76, ILE79, ARG80, ASP82, ASP104 and PHE103. (B) TSA had better interactions with HDAC2 compared to other HDAC of Class 1 by interacting with HIS183, HIS146, LEU144, ASP104, CYS156 and MET35. (C) Interaction between TSA and HDAC3 was by formation of bonds with aminoacid residues like HIS17, TYR331, VAL300, and ARG301. (D) TSA interacted with HDAC8 by linking with PHE152, GLY151, PHE208, HIS180 and TYR306. (E) TSA interacted with Class II HDAC-4 by forming bonds with HIS54 residue. (TIF) [file pone.0186208.s001.tif]

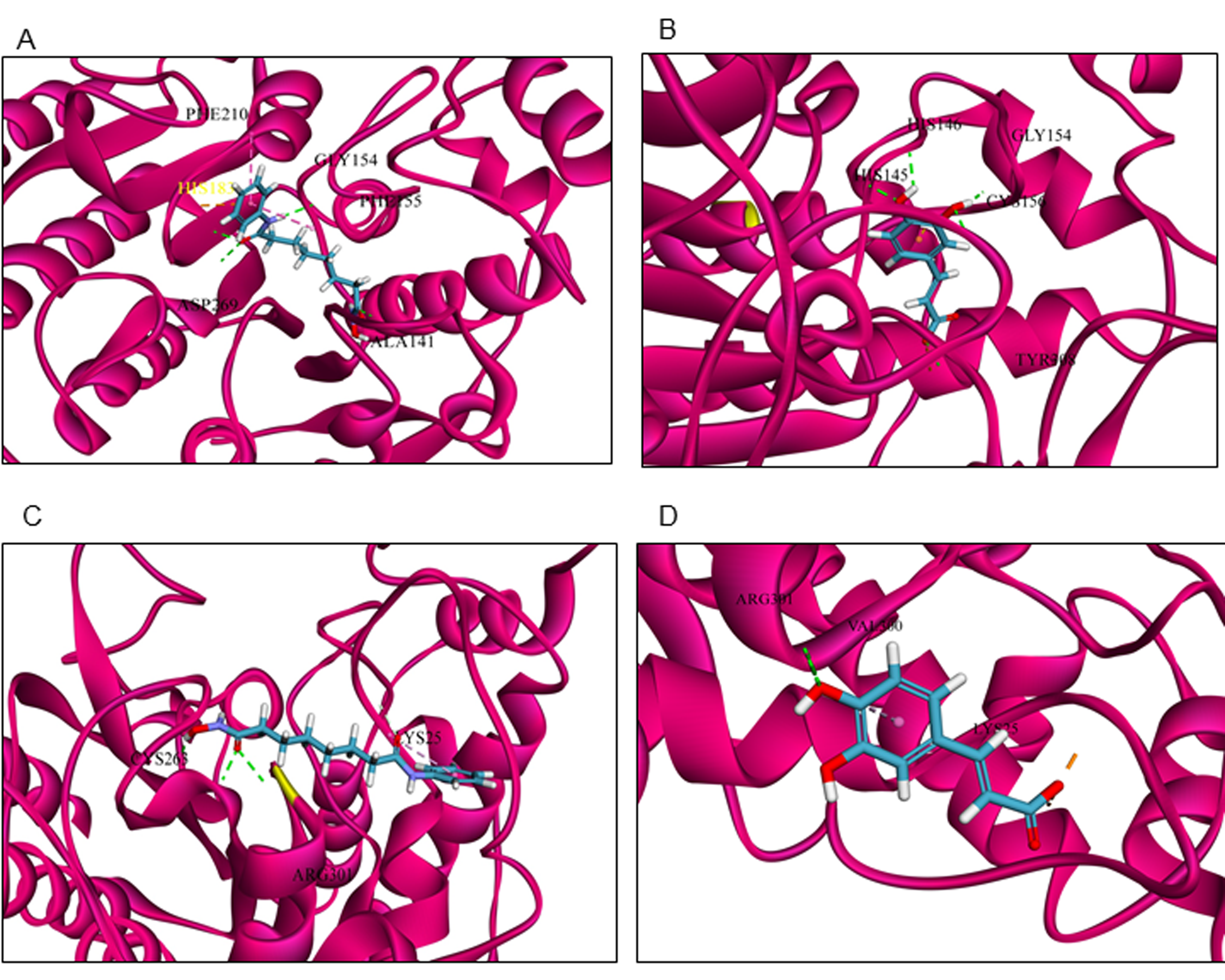

Supplement: S2 Fig — (A): HDAC inhibitor SAHA interacted with HDAC2 with key amino acid residues such as ALA141, HIS 146, HIS183, ASP269, PHE155, PHE210 (B) DHCA bound to SAHA binding site at HDAC2 by interacting with HIS145, HIS146, TYR308, GLY154, CYS156 (C) SAHA interacted with ARG301 and CYS263 of HDAC3 (D): DHCA also interacted with HDAC3 at ARG301, ARG265 and LYS25. (TIF) [file pone.0186208.s002.tif]

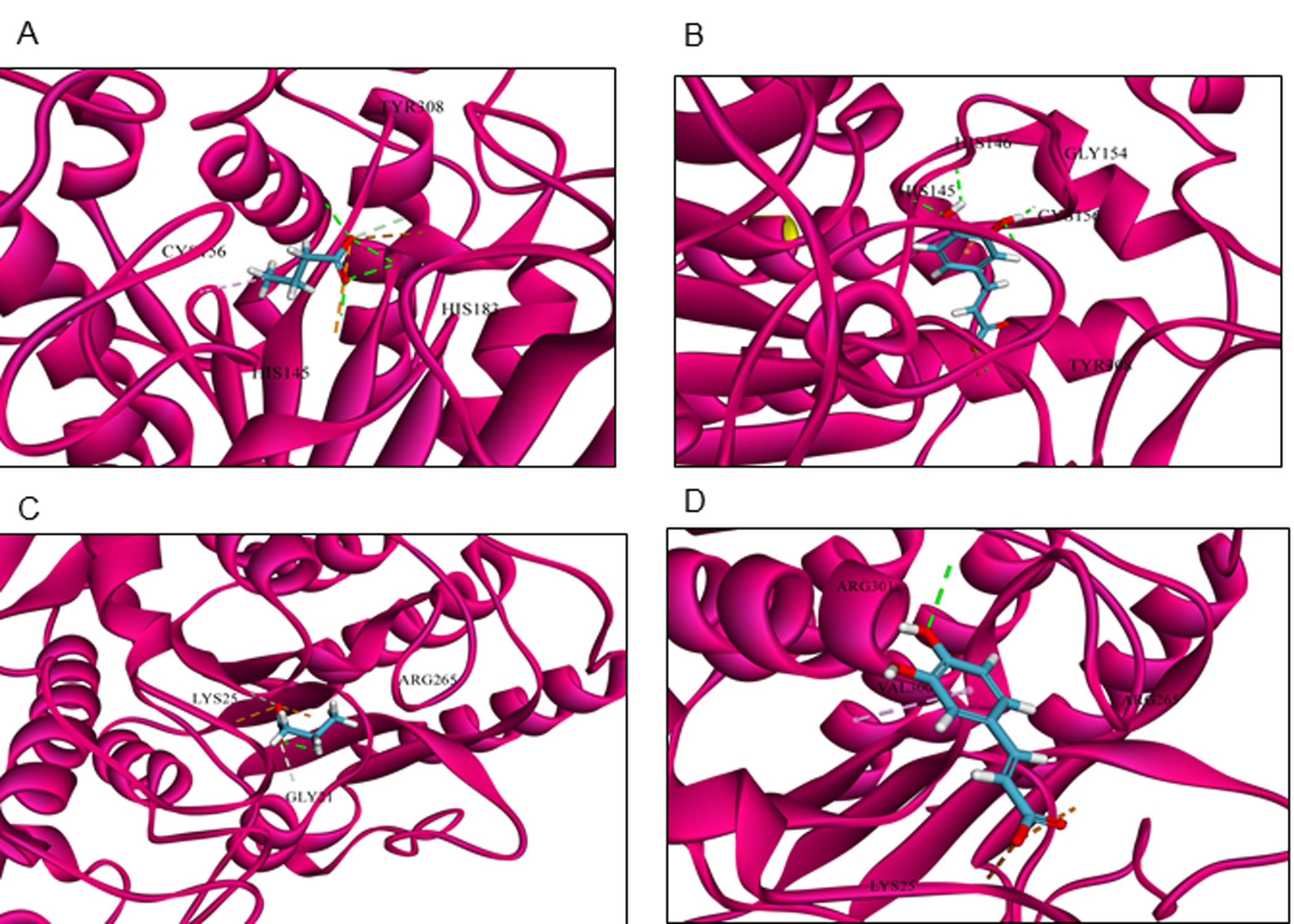

Supplement: S3 Fig — (A): Sodium butyrate a known HDAC inhibitor interacted with HDAC2 with key amino acid residues such as HIS145, HIS183 and TYR 308 (B) DHCA interacted with HDAC2 at sodium butyrate binding site by forming bonds with HIS145, HIS146, TYR308, GLY154, CYS156 (C) Sodium butyrate interacted with ARG265 and LYS25 of HDAC3 (D): DHCA also interacted with HDAC3 at ARG301, ARG265 and LYS25. (TIF) [file pone.0186208.s003.tif]

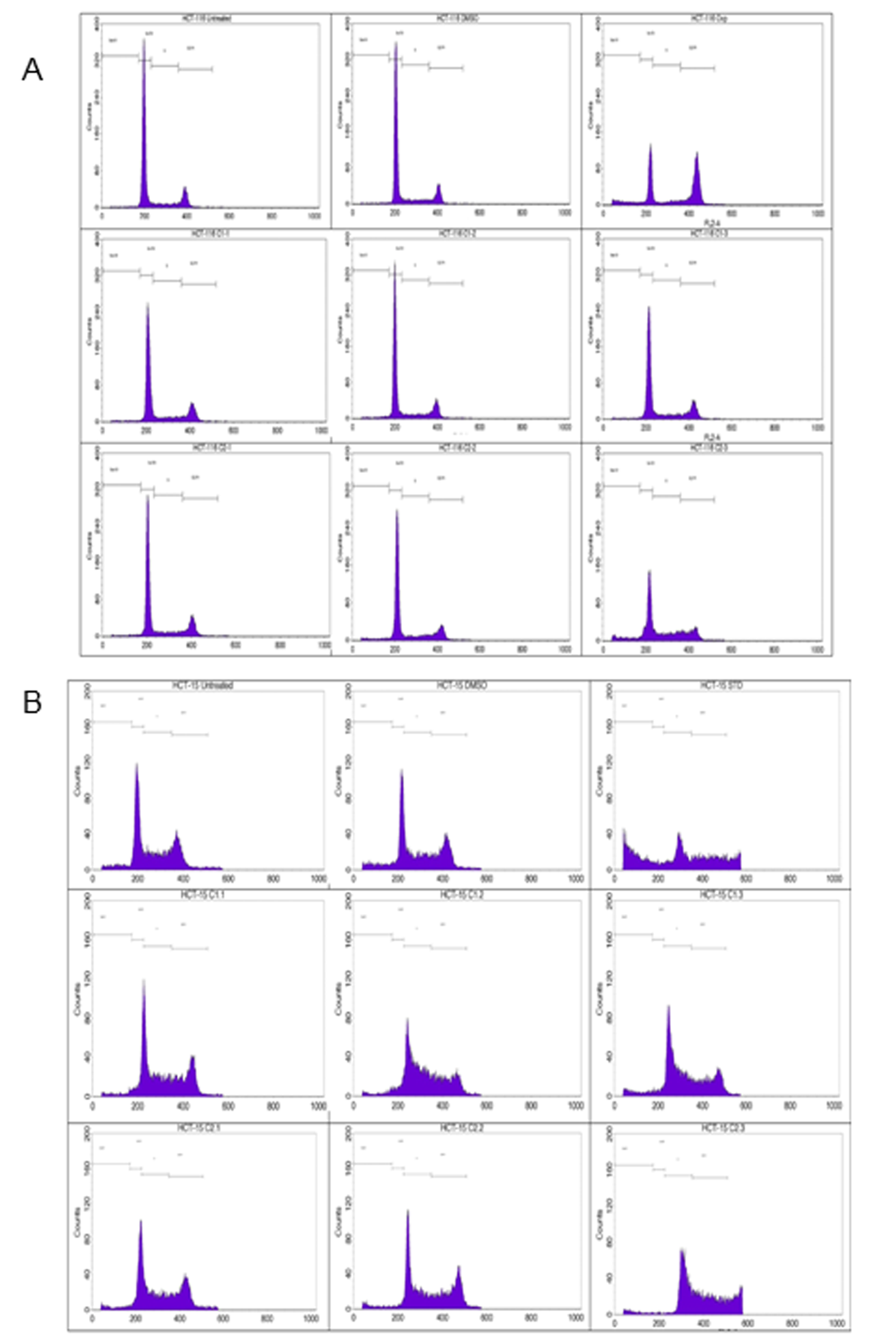

Supplement: S4 Fig — (A): DHCA arrested HCT-116 cell lines in G2/M phase ion a dose dependent manner when compared to untreated and vehicle treated cells at 48h. (B) DHCA also inhibited HCT-15 cells in a dose dependent fashion at 48h. (TIF) [file pone.0186208.s004.tif]
